# Low Back Pain in Primary Care: A Description of 1250 Patients with Low Back Pain in Danish General and Chiropractic Practice

**DOI:** 10.1155/2014/106102

**Published:** 2014-11-04

**Authors:** Lise Hestbaek, Anders Munck, Lisbeth Hartvigsen, Dorte Ejg Jarbøl, Jens Søndergaard, Alice Kongsted

**Affiliations:** ^1^Department of Sports Science and Clinical Biomechanics, University of Southern Denmark, 5230 Odense, Denmark; ^2^Nordic Institute of Chiropractic and Clinical Biomechanics, Campusvej 55, 5230 Odense, Denmark; ^3^Audit Project Odense, Institute of Regional Health Services Research, University of Southern Denmark, 5000 Odense C, Denmark; ^4^Research Unit of General Practice, Institute of Public Health, University of Southern Denmark, 5000 Odense C, Denmark

## Abstract

*Study Design*. Baseline description of a multicenter cohort study. *Objective*. To describe patients with low back pain (LBP) in both chiropractic and general practice in Denmark. *Background*. To optimize standards of care in the primary healthcare sector, detailed knowledge of the patient populations in different settings is needed. In Denmark, most LBP-patients access primary healthcare through chiropractic or general practice. *Methods*. Chiropractors and general practitioners recruited adult patients seeking care for LBP. Extensive baseline questionnaires were obtained and descriptive analyses presented separately for general and chiropractic practice patients, Mann-Whitney rank sum test and Pearson's chi-square test, were used to test for differences between the two populations. *Results*. Questionnaires were returned from 934 patients in chiropractic practice and 319 patients from general practice. Four out of five patients had had previous episodes, one-fourth were on sick leave, and the LBP considerably limited daily activities. The general practice patients were slightly older and less educated, more often females, and generally worse on all disease-related parameters than chiropractic patients. All differences were statistically significant. *Conclusions*. LBP in primary care was recurrent, causing sick leave and activity limitations. There were clear differences between the chiropractic and general practice populations in this study.

## 1. Introduction

Low back pain (LBP) is the leading cause for years lived with disability worldwide [1] and the cost for society is huge. In Denmark, the accumulated societal costs related to back pain were estimated at 2.8 billion US dollar in 2005, almost equivalent to one percent of the NGP [2], with the vast majority of the costs being spent on the minority of the cases that become chronic. Therefore, focus has centered on chronic patients in secondary and tertiary care settings whereas patients in primary healthcare have received less attention. This is in spite of almost all initial consultations occurring in primary care and musculoskeletal problems being one of the most common reasons for consultation [3]. Among politicians and health care planners, there is a growing awareness of the important role of primary care as the “first line of defense” in reducing chronicity and long-term disability. This has led to increased demands for implementation of clinical guidelines, quality assessment and assurance, consensus about “best treatment,” and so forth.

To optimize standards of care in the primary care sector, detailed knowledge of the patient population regarding demographics and socioeconomic factors as well as disease-specific characteristics can help in making informed decisions, but so far the LBP-population in different primary care settings has been poorly described. In Denmark, about one-third of all healthcare-seeking LBP-patients access primary healthcare through chiropractic practice [4] and most others via general practice. Musculoskeletal disorders are the reason for 14% of all consultations in Danish general practice and are thus the most common complaint of all consultations [3]. Among the musculoskeletal disorders, back pain is the most common, constituting at least 3.5% of total visits (personal communication, Grethe Moth, Research Unit for Primary Care, Aarhus, Denmark) and in chiropractic practice, LBP is the reason for 49% of consultations [5]. If these two patient populations significantly differ, various guidelines, stratification tools, prediction rules, and so forth may not have the same relevance in both types of practices. This is exemplified by the STarT Back Screening Tool, which in the UK has been shown to have good prognostic value in general practice [6] but not in chiropractic practice [7]. Hence, it is necessary to investigate whether there are differences between populations presenting with similar symptoms but in different types of practice, since research results might not be transferable between different practice types and potential differences should be taken into account for allocation of resources as well as for targeting guideline recommendations.

Most studies investigate monoprofessional clinical samples but to get a more complete picture of the Danish primary care LBP-population, patients from both chiropractic and general practice should be included. Therefore, the objectives of this study were (1) to describe patients with LBP in chiropractic and general practice in Denmark with respect to disease specific and demographic factors and (2) to investigate possible differences between the two populations.

## 2. Materials and Methods

### 2.1. The Multicenter Cohort Study of Low Back Pain Patients

This study reports on baseline data from a prospective cohort study aimed at identifying course patterns, subgroups, and risk factors in LBP-patients. The study included LBP-patients from both general practice and chiropractic practice to reflect patients in primary healthcare. In connection with a consultation for LBP, patients were examined and filled out baseline questionnaires. Clinical examination and followup procedures were not part of the present study and have been described elsewhere [8, 9].

### 2.2. Recruitment from Chiropractic Practice

Chiropractors from 17 chiropractic clinics in the research network of the Nordic Institute for Chiropractic and Clinical Biomechanics [8] agreed to recruit consecutive patients with LBP from September 2010 till January 2012. The primary objective of this initiative was to investigate the prognosis of patients treated by chiropractors for a new episode of back pain. Participating chiropractors attended a one-day course about the study procedures and a research assistant visited the clinics to confirm procedures before the study began.

Prior to inclusion, patients received written and verbal information about the study. The patients who decided to participate completed the baseline questionnaire in the reception area before the first consultation and returned in a closed envelope to the clinic secretary who sent it to the Nordic Institute of Chiropractic and Clinical Biomechanics.

### 2.3. Recruitment from General Practice

All 800 GPs in the Region of Southern Denmark were invited to participate in a quality development initiative by the Audit Project Odense (APO) [10]. The objective of this initiative was to evaluate the use of the STarT back screening tool [11], implementation of electronic data capture, and the establishment of a cohort of patients with LBP to be followed prospectively. Eighty-eight GPs agreed to participate. During 10 weeks of 2011 they registered 421 patients who consulted for LBP (ICPC-2 code of L02, L03, and L86). If a patient consulted more than once during the registration period, only the first visit was registered. Following the consultation, the patient was given an envelope containing information about the prospective study, an invitation to participate, a baseline questionnaire, and a prepaid return-envelope. If the patient decided to participate in this study, the baseline questionnaire was completed at home after the consultation and sent to the Nordic Institute of Chiropractic and Clinical Biomechanics. Some patients returned questionnaires although the GP did not register any clinical data.

To increase the size of the cohort, the 12 GPs who had recruited most patients during the quality development initiative and 18 other GPs known to have an interest in LBP were asked to do a supplementary recruiting for the remainder of 2011.

*Inclusion Criteria for Both Chiropractic and General Practice*

- LBP with or without radiating pain,
- 18–65 years of age,
- access to a mobile phone.

*Exclusion Criteria*

- Pregnancy,
- suspicion of serious pathology,
- inability to read and write Danish.

*Additional Exclusion Criteria for Chiropractic Practice*

 Consulting any type of healthcare provider for LBP the previous three months.

### 2.4. Variables

Data were based on patient-reported baseline questionnaires. The nonclinical data used for this report included age, gender, BMI, smoking, education, and health insurance. The disease specific information was pain intensity (back and leg) reported on an 11-point numerical rating scale (NRS) [12], pain duration, number of previous episodes, sick leave, recovery expectations, disability as measured by the Roland Morris Disability Questionnaire [13], and risk of a poor prognosis estimated by means of the STarT back screening tool [14]. Furthermore, depression was measured by the Major Depression Index [15] and general health assessed by the visual analog scale of the EQ-5D [16].

### 2.5. Analyses

Analysis of nonresponders was not possible for chiropractic patients, since there was no information on LBP-patients not returning questionnaires. For the patients in general practice, responders to the baseline questionnaire were compared to the patients registered in the quality development initiative who did not return the questionnaires, with regard to age, gender, duration of LBP, previous episodes of LBP, and the presence of leg pain.

No imputations of missing values were performed. The proportional score for the Roland Morris disability questionnaire was calculated for all respondents answering at least 17 of the 23 items, as suggested by Kent and Lauridsen [13]. The total scores for the Major Depression Inventory and the Fear Avoidance Beliefs Questionnaire (physical activity and work) were calculated for those with complete responses.

Descriptive analyses based on the two populations combined are not likely to represent the LBP-population in primary care in general, because the relative size of the two populations does not reflect reality. Therefore, all descriptive analyses are presented separately for general and chiropractic practice patients. Proportions are presented with 95% confidence intervals (CI) and medians with interquartile ranges (IQR). Data were not normally distributed and therefore Mann-Whitney rank sum test was used to test for similar distributions between the two samples for the continuous variables, and Pearson's chi-square test was used to test for differences between the two populations for categorical and dichotomous variables.

### 2.6. Post Hoc Analyses

Due to a large amount of nonresponders to the baseline questionnaire in general practice, we decided to do a crude sensitivity analysis of the difference between patients in general practice and chiropractic practice. To estimate the least possible difference between the populations, we assumed that all nonresponders in general practice resembled patients in chiropractic practice. Based on this, proportions of patients in the worst category were estimated with 95% CI for the noncontinuous variables (low education, duration of at least one month, 3 or more previous episodes, sick leave at least one week, presence of leg pain, and high risk group according to the STarT back screening tool categorization) and the CIs were compared to those of the chiropractic population.

All analyses were done with Stata 11.1 (StataCorp LP).

## 3. Results

Valid patient questionnaires were received from 934 chiropractic patients. General practitioners included 345 patients in the quality development initiative, of whom 206 (60%) agreed to participate in the follow-up study and thus returned the baseline questionnaire. Further 113 patients from general practice returned questionnaires but were not registered in the quality development initiative, resulting in 319 questionnaires from general practice patients.

*Nonresponders in General Practice*. The 139 nonresponders tended to have a less severe profile, based on potential prognostic factors registered by the general practitioners, than responders: they were younger and more often males and had less leg pain, less sick leave, fewer previous episodes, and a shorter duration. However, the observed differences were small and not statistically significant.

*Nonclinical Characteristics of LBP-Patients in Primary Healthcare*. The majority of the patients were between 35 and 55 years of age with the general practice sample being slightly older than those from chiropractic practice (*P* < 0.01). There were statistically significantly more females, more smokers, and more patients with lower education in general practice than in chiropractic practice, but the BMI was similar in the two populations and there was no difference in the proportion with private insurance (Table 1).

*Disease-Specific Characteristics of LBP-Patients in Primary Healthcare*. Only 16% (95% CI: 14%–18%) of the whole study population presented with a first episode of LBP and 52% (95% CI: 49%–55%) reported more than three previous episodes. Twenty-six percent (95% CI: 24%–29%) were sick listed and 5% (95% CI: 4%–6%) of these had been so for more than a week. There was a large proportion of patients who reported pain radiating to the leg: 58% (95% CI: 55%–61%) of patients in chiropractic practice and 68% (95% CI: 63%–74%) in general practice. Nevertheless, recovery expectations were generally high (median 9 (IQR 7–10) in chiropractic practice and 6 (IQR 3–9) in general practice) and expectations below 2 on a 0–10 scale were only seen in 54 cases in the combined cohort (2%; 95% CI: 1%–3% in chiropractic practice and 11%; 95% CI: 8%–15% in general practice).

**Figure 1:**
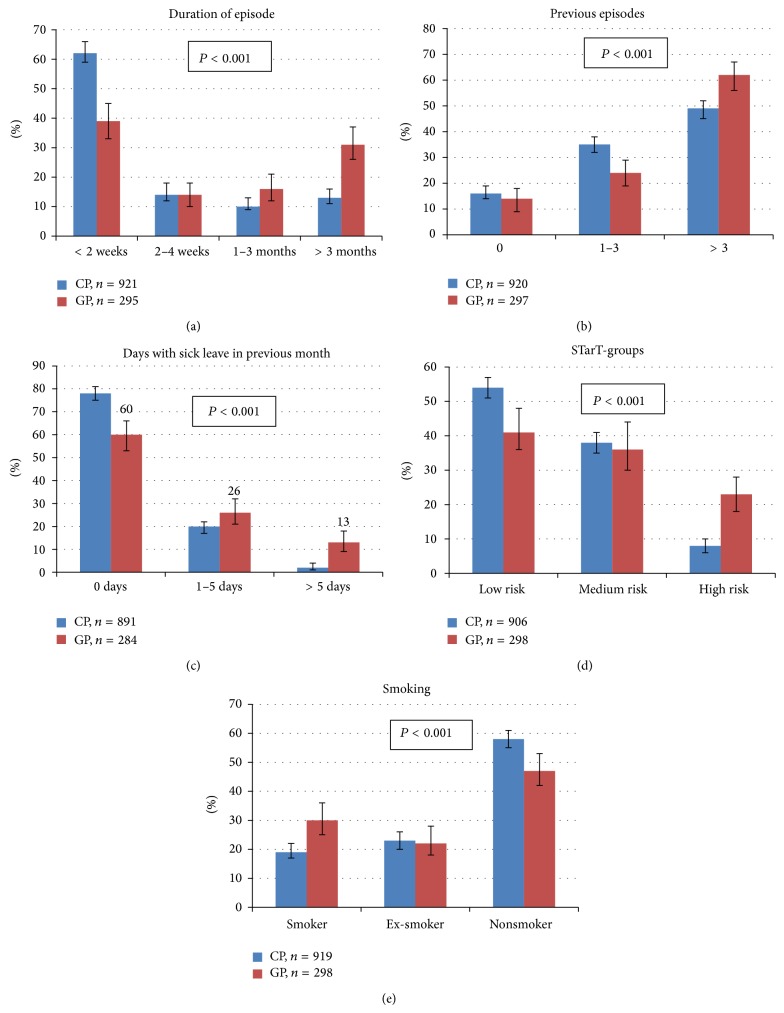
Distribution of the categorical variables for LBP-patients in chiropractic practice (CP) and general practice (GP), respectively. The variables are duration of the present episode, number of previous episodes, days with sick leave in the previous month, risk of chronicity as categorized by the STarT back screening tool, and smoking status. Percent with 95% confidence intervals. *P* values represent significance levels for the differences between the two populations and are based on Pearson's chi-square test.

All the disease specific parameters showed a statistically significant difference between general and chiropractic practice. Patients in general practice were generally more severely affected. They had higher pain intensity (mainly for leg pain), longer pain duration, more previous episodes, more sick leave, more activity limitation on the disability scale, slightly higher level of depression, slightly more fear-avoidance beliefs, and a poorer self-reported general health. Details are presented in Table 2 and Figure 1.

There was also a substantial difference in the proportion stratified to the high-risk category according to the STarT back screening tool (Figure 1).

### 3.1. Post Hoc Analysis

In the hypothetical situation where the missing data are imputed to minimize the difference between settings, that is, all nonresponders in general practice resemble patients in chiropractic practice, there would still be differences in the observed direction between the two populations for all the investigated variables. Only the difference in gender and leg pain did not reach statistical significance.

## 4. Discussion

Our results show that LBP is not negligible in primary healthcare and support previous studies suggesting that LBP should not be seen as benign and self-limiting [17, 18]. Although chiropractic patients, who were generally less severely affected than patients from general practice, were overrepresented in our cohort, four out of five had had previous episodes, about half of them had had more than three episodes, and one-fourth were on sick leave. Furthermore, judging from the scores on the Roland Morris Disability Questionnaire, the LBP considerably limits daily activities and almost one-third of the patients in general practice were in the group with high risk of chronicity according to the STarT back screening tool. Considering that musculoskeletal disorders are the most common somatic reason for disability pension [19, 20], a strong and focused effort should be implemented in primary healthcare to reduce chronicity and disability.

The manifest high frequency of recurrences supports findings from research over the past decade which has made it clear that LBP, as well as other types of musculoskeletal disorders, should be considered in a lifetime perspective rather than considering each episode as a separate entity [21]. This makes the need for good and evidence-based treatment early in the course even more evident and this has to be initiated in primary care. However, the results of this report illustrate the importance of conscientious and detailed reporting of research and the need for caution when transferring results between settings.

Clear differences between the chiropractic and general practice populations were observed in this study. The general practice patients were slightly older and less educated, there were more females and more smokers, their leg pain was generally worse, and they scored higher on the depression index than patients in chiropractic practice. Although the differences were statistically significant, some of the observed differences were small in size and may not be of clinical relevance. Nevertheless, especially the large difference in the proportion of high-risk patients, when stratified according to the STarT back screening tool, could have implications for health care planning [6] with more emphasis on psychosocial factors in settings with many high-risk patients.

It should be recognized that responders and nonresponders in general practice differed in the same direction as general practice and chiropractic patients, and therefore the reported difference between the two settings might be overestimated. However, the magnitude of the difference between the two populations was considerably larger than the difference between responders and nonresponders in general practice, indicating a real difference between populations. Furthermore, although the sensitivity analysis can only provide an indication of a scenario resembling the smallest possible difference, it did support the findings of the main analyses, since most differences were still statistically significant. Therefore, the difference between the two populations seems to be robust. This is also in accordance with a recent description of Danish chiropractic patients which demonstrated that the average chiropractic patient was better educated and younger than the average Danish population [22].

There could be several reasons for the found differences between the populations, and they may vary between countries [23] although similar differences have also been demonstrated in the USA [24]. Since consultations in general practices are free of charge in Denmark, economic considerations are likely to influence choice of care provider, which could partly explain the higher education and the predominance of males among the chiropractic patients, which is in line with previous surveys [5, 22]. Apart from the economic considerations, there are no differences in access between the two types of care. There is a wide geographical spread of both groups across the country and no referral or approval is needed for either. However, several other factors may influence choice of healthcare provider: comorbidities might tempt patients to choose their general practitioner, who is acquainted with the previous health history; social factors are likely to play a role, since recommendations from family and other acquaintances probably influence the choice of nonmedical treatment; patients with higher education could be more motivated to return to work early and are therefore more active care seekers; considering the high recovery expectations in chiropractic practice, differences in illness perception might also play a role; and finally, the choice of provider might have historical reasons since chiropractic is a relative newcomer in health care compared with medicine. Whatever the reason, the consequences of the different choices of care should be investigated and the gained knowledge included in a reconsideration of the present organization of the health care system to optimize the use of both financial and human resources [25, 26].

The study had obvious weaknesses: there was no data on nonresponders in chiropractic practice and the timing was different in the two settings, since chiropractic patients filled out the questionnaire before the first consultation and general practice patients after. Both of these shortcomings hamper the validity of the comparison between chiropractic and general practice patients. Furthermore, as in many other studies, both types of practitioners were asked to include “consecutive patients,” but the large variation in recruited patients per practitioner indicates that this did not happen. There was no recording of all patients during the sampling period and therefore differences between invited and not invited could not be explored, and thus the practitioners' conscious or subconscious selection bias is unknown.

Nevertheless, for the descriptive purpose of this study, it was a conclusive strength that patients from both settings which are entrance pathways to the Danish health care system for patients with LBP were included. Furthermore, the sample is relatively large with an extensive baseline questionnaire, allowing descriptions of many parameters.

## 5. Conclusions

Four out of five patients had had previous episodes, about half of them had more than three, one-fourth were on sick leave, and the LBP considerably limited daily activities. This shows that the severity of LBP in primary care is not negligible and illustrates that LBP should not be considered as benign and self-limiting. Thus, to reduce the long-term consequences, a rational and coordinated effort in primary care is called for. The general practice patients were slightly older and less educated, there were more females, and their LBP was generally worse than chiropractic patients. Further investigations into the consequences of this apparent self-selection are needed to consider the best way of distributing the responsibility for these patients to optimize the use of health care resources. The clear differences between the chiropractic and general practice populations in this study illustrate that research results are not directly transferable between different practice types, despite similar complaints, and potential differences should be taken into account for allocation of resources as well as for targeting guideline recommendations.

## Figures and Tables

**Table 1:** Nonclinical variables from 934 LBP patients from chiropractic practice and 319 from general practice. GP: general practice; CP: chiropractic practice.

	CP	GP	*n*, CP/GP	*P*
Age, median years (IQR)	43 (34–53)	46 (38–54)	934/319	<0.004^#^
Gender, % female (95% CI)	45 (42–48)	55 (50-51)	934/319	0.001^*^
BMI, median (IQR)	26 (23–28)	26 (23–29)	915/286	0.080^*^
Education, % low^†^ (95% CI)	46 (43–49)	60 (54–66)	919/297	<0.001^*^
Private insurance, % yes (95% CI)	38 (35–42)	36 (31–42)	882/294	0.721^*^

^#^Mann-Whitney rank sum test for continuous variables to test for similar distributions between the two samples. ^*^Pearson *χ* ^2^ test for difference.

^†^high school or less.

**Table 2:** Continuous, clinical variables from 934 LBP patients from chiropractic practice and 319 from general practice. GP: general practice; CP: chiropractic practice. Median (IQR).

Variable	Median (IQR) CP	Median (IQR) GP	*n* CP/GP	*P* ^*^
Intensity LBP (0–10) (0 = no pain)	7 (5–8)	7 (6–8)	896/293	0.002

Intensity leg pain (1–10)^**^ (1 = minimum detectable pain)	4 (2–6)	6 (3–8)	896/240	<0.001

Patient expectations^1^ (1–10) (1 = no recovery)	9 (7–10)	6 (3–9)	926/295	<0.001

MDI^2^ (0–60) (0 = no depressive symptoms)	6 (3–11)	9 (5–18)	924/294	<0.001

RM^3^ (0–100) (0 = no limitations)	52 (35–70)	61 (39–78)	925/301	<0.001

FABQ-a^4^ (0–24) (0 = no fear and avoidance beliefs)	13 (9–17)	14 (10–18)	893/289	0.009

FABQ-w^5^ (0–42) (0 = no fear and avoidance beliefs)	11 (6–20)	14 (7–22)	801/229	<0.001

Self-perceived general health^6^ (0–100) (0 = worst possible health)	70 (60–80)	60 (40–78)	908/238	<0.001

^*^Mann-Whitney rank sum test for continuous variables to test for similar distributions between the two samples.

^**^Only patients with >0 on the VAS scale included (*n* = 520/166)

^1^“What do you think the chances are that you have recovered completely in three months?,” ^2^Major Depression Inventory, ^3^Roland Morris Disability Questionnaire, ^4^Fear Avoidance Beliefs Questionnaire, physical activity, ^5^Fear Avoidance Beliefs Questionnaire, work, ^6^Quality of Life (Euroqol 5D).
